# EEG Based Classification of Long-Term Stress Using Psychological Labeling

**DOI:** 10.3390/s20071886

**Published:** 2020-03-29

**Authors:** Sanay Muhammad Umar Saeed, Syed Muhammad Anwar, Humaira Khalid, Muhammad Majid, Ulas Bagci

**Affiliations:** 1 Department of Computer Engineering, University of Engineering and Technology, Taxila 47050, Pakistan; sanay.muhammad@uettaxila.edu.pk (S.M.U.S.); m.majid@uettaxila.edu.pk (M.M.); 2 Department of Software Engineering, University of Engineering and Technology, Taxila 47050, Pakistan; 3 Department of Computer Science, University of Central Florida, Orlando, FL 32816, USA; bagci@ucf.edu; 4 Department of Psychology, Benazir Bhutto Hospital, Rawalpindi 46000, Pakistan; merimalik18@gmail.com

**Keywords:** long-term stress, electroencephalography, machine learning, perceived stress scale, expert evaluation

## Abstract

Stress research is a rapidly emerging area in the field of electroencephalography (EEG) signal processing. The use of EEG as an objective measure for cost effective and personalized stress management becomes important in situations like the nonavailability of mental health facilities. In this study, long-term stress was classified with machine learning algorithms using resting state EEG signal recordings. The labeling for the stress and control groups was performed using two currently accepted clinical practices: (i) the perceived stress scale score and (ii) expert evaluation. The frequency domain features were extracted from five-channel EEG recordings in addition to the frontal and temporal alpha and beta asymmetries. The alpha asymmetry was computed from four channels and used as a feature. Feature selection was also performed to identify statistically significant features for both stress and control groups (via *t*-test). We found that support vector machine was best suited to classify long-term human stress when used with alpha asymmetry as a feature. It was observed that the expert evaluation-based labeling method had improved the classification accuracy by up to 85.20%. Based on these results, it is concluded that alpha asymmetry may be used as a potential bio-marker for stress classification, when labels are assigned using expert evaluation.

## 1. Introduction

The response of the human body to a demand for change is considered as stress [1]. A balance exists between the sympathetic and parasympathetic arms of the autonomic nervous system in healthy people. A fight-or-flight response is invoked when there is an exposure to a threatening situation. Daily routine stress does not pose any danger to life, however, the fight-or-flight response may still be invoked. A persistence of this short-term stress for a longer duration can cause long lasting effects on the neurology of an individual and may give rise to depression [2]. Long-term stress is a better predictor of depressive symptoms as compared to short-term stress [3]. Long term stress is considered a risk factor for many health conditions such as cardiovascular diseases [4,5].

The prevention of the onset of depression requires a timely detection of long-term stress symptoms. Conventional psychological methods and analysis of hormones such as cortisol and alpha-amylase are widely used in long-term stress studies [6]. These methods are practical but they are affected by various factors, such as language and objectivity. For instance, the Perceived Stress Scale (PSS) is a widely used questionnaire to measure the level of chronic stress, validated extensively across diverse samples [7]. Though in general, a self-administered checklist cannot equal the precision of an interviewer trained to elicit aspects of events critical to examine stress. Such interviews have shown to provide substantially better information in comparison to relatively unassisted self-reporting mechanisms [8]. Respondents have been found to report minor or positive events in response to questions designed to elicit negative and undesirable events [9].

Psychological methods alone are not enough to assess stress-related conditions [10]. Stress can be quantified objectively from bio-markers like electroencephalography (EEG), galvanic skin response, and electrocardiography [11]. Recently, wearable systems were developed that can record electro-physiological signals (such as EEG and heart rate variability) to detect acute stress [12]. EEG is one of the most common source of information for studying brain function [13,14,15,16,17]. The oscillations generated by the variation of electric potential in the brain are recorded using low resistance electrodes placed on the human scalp [18]. It is a widely used noninvasive method due to its excellent temporal resolution, ease of use, and low cost. EEG signals are categorized by their frequency bands including delta, theta, alpha, beta, and gamma. Each frequency band can be used as a discriminating feature for different brain states [19]. There are methods reported in literature to quantify human acute stress in response to induced stressors using EEG signal recordings. In comparison, the classification of long-term or chronic stress using EEG has not been widely assessed.

### Our Contributions

In this study, the problem of long-term human stress recognition is addressed by using PSS labels and expert evaluation, which has not been explored before. We have hypothesized that wearable sensors (such as those for recording brain activity using EEG electrodes) can be used for identifying chronic stress, without inducing stress using a stimulus. To this end, our experiments have shown that involving a psychology expert for labeling stressed and control subjects is beneficial for such a classification. It is important to note here that we did not use any stimulus in our study to induce stress so that this system can be administered for detecting stress in daily life routine. Two groups of participants were considered including the stressed group and the control group. A total of forty five different features were extracted from EEG signals in frequency domain to classify these two groups. Discriminating features were selected using a statistical significance test. Five different machine learning classifiers including support vector machine (SVM), Naive Bayes (NB), K-nearest neighbor (KNN), logistic regression (LR), and multi-layer perceptron (MLP) were used to classify human stress using the selected features. Due to limitations of the data size and the noisy nature of the signals, deep-learning-based systems were not suitable for the task at hand. Therefore, we concentrated on machine learning classifiers that are more suitable for the task that we target to solve. The summary of our findings in this study is as follows:

1. We used EEG signals acquired from 33 participants in closed eye conditions using a five-channel EEG headset for long term stress classification (no stimuli used to induce stress) and found that among different feature, three frequency domain features were statistically significant in stress and control groups.
2. To the best of our knowledge, this is the first that the stress level of participants was labeled by a psychology expert in an EEG-based study. We showed its feasibility with a validated set of experiments.
3. The conventional machine learning classifiers suite well to long-term human stress classification and give better performance using psychological expert labeling.

The rest of the paper is organized as follows: Section 2, describes the related work. Section 3 presents the proposed methodology including data collection, feature extraction, and classification algorithms. Section 4 presents the results and a comparison with previously reported studies. Finally, the conclusion of the study is given in Section 5.

## 2. Related Work

Hemispheric specialization is a major concern in neuro-physiological research. Generally, a healthy brain at rest has a fairly balanced level of activity in both hemispheres of brain [20]. The left hemisphere is associated with the processing of positive emotions, while the right hemisphere is associated with the processing of negative emotions [21]. The extent of asymmetry has been suggested to vary under conditions of chronic stress [22]. Frontal asymmetry is highly related to post-traumatic stress disorder (PTSD) [23]. The results in [24], have shown that major depression disorder (MDD) group is significantly right lateralized relative to controls, and both MDD and PTSD displayed more right- than left-frontal activity.

Recently, the feasibility of using EEG in classifying multilevel mental stress has been demonstrated [19], where alpha rhythm at the right pre-frontal cortex was suggested as a suitable bio-marker. A machine learning framework using EEG signals was proposed in [25], where stress was induced by using the Montreal imaging stress task (MIST), and SVM, NB, and LR classifiers were used to classify the stress level of participants. The EEG of participants in resting-state was recorded under negative, positive, and neutral stimulus using soundtracks from the international affective digitized sounds (IADS-2) dataset [26]. Stress detection based on frontal alpha asymmetry was performed using the DEAP dataset, and classification was performed using SVM, KNN, and fuzzy KNN [27]. In [28], a mobile EEG was used to assess stress in humans using EMOTIV EPOC headset in an out-of-lab environment. In an EEG based study, 11 participants were analyzed for the identification of long-term stress [10], including seven mothers of children with mental disability (stress group) and four mothers of healthy children (control group).

A variant of the trier social stress task (TSST) was used to assess stress in 49 participants [29]. Samples of the salivary cortisol and resting state EEG based alpha asymmetry were assessed before and after performing TSST. The frontal and parietal alpha asymmetry was used to classify depression in elderly people [30]. The correlation between frontal and parietal alpha asymmetry, the geriatric depression scale, and the mini mental state examination were analyzed. A high beta activity at the frontal and occipital lobes was observed on the visual input of negative images [31]. The frontal theta activity was shown to decrease due to a stressful mental arithmetic task [32]. In [33], low beta waves in closed eye condition were found to be a strong predictor of perceived stress, where PSS score was predicted by using multiple linear regression. The pre-frontal relative gamma power i.e., the ratio of gamma band and slow brain rhythms, was proposed as a bio marker for identification of stress [34,35].

The related studies presented here can be grouped as either short-term or long-term stress assessment. Short-term stress is measured using a stress eliciting task, while long-term stress is measured without performing any additional mental task. Different techniques have been adopted to measure stress, but most of these techniques require human intervention. Among different physiological measures, EEG has the potential to be used as a measure of stress in daily life. This is due to the fact that EEG headsets are becoming commercially available for observing brain activity in an easy to wear and cost effective manner. The proposed study uses EEG signals acquired with a commercially available EEG headset to identify baseline or long-term stress without relying on stress-inducing tasks.

## 3. Methodology

**Figure 1:**
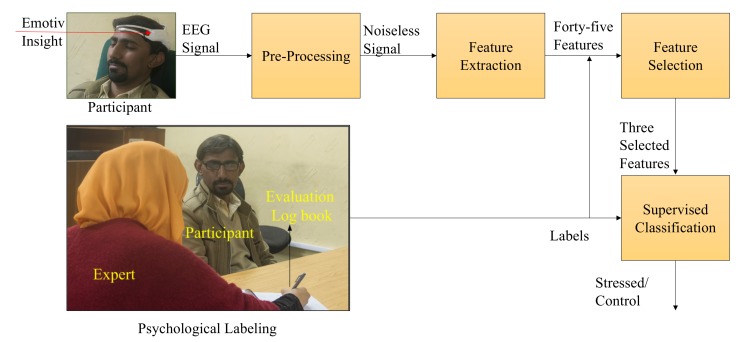
The proposed methodology for long-term human stress classification.

We devised a supervised machine learning model for the classification of human stress (Figure 1). A total of 33 volunteers participated in this study. The resting state EEG data for each participant were acquired using an EMOITV Insight headset (https://www.emotiv.com/insight/) in a closed eye condition for three minutes. After EEG signal recording, participants were asked to fill in the PSS-10 questionnaire followed by an interview with the psychology expert. The average time for the interview was 25 min. Based on the PSS scores and interview, the psychology expert grouped each participant in either the stress or the control group.

**Figure 2:**
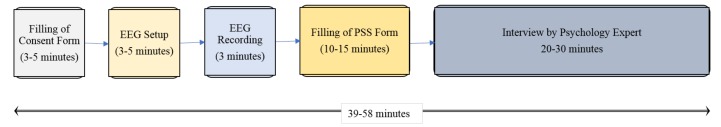
Experimental sequence and the data acquisition process.

The recorded EEG signals were made noise free in the pre-processing stage. Neuro-physiological features including alpha (α), low beta ($\beta_{l}$), beta (β), gamma (γ), delta (δ), theta (θ), and relative gamma (RG) power were extracted from the signals at each electrode. Frontal and temporal alpha and beta asymmetries, and alpha asymmetry was calculated from these features. Five supervised machine learning algorithms (SVM, NB, KNN, LR, and MLP) were used to classify human stress. Two different labeling methods were used, including the perceived stress scale and expert evaluation, where the PSS and interview scores were simultaneously used. A detailed description of these methods is presented in the following subsections. flow of events during the data acquisition process is shown in Figure 2.

### 3.1. Data Acquisition

**Figure 3:**
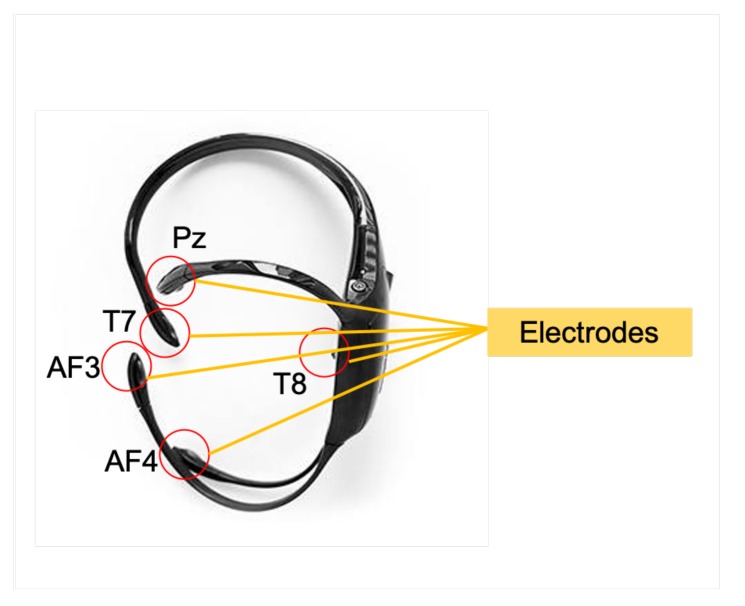
The Emotiv headset with five electrodes marked at positions AF3,AF4,T7,T8,andPz.

All EEG recordings were performed in a noise free lab using the EMOTIV Insight headset, which records brainwaves and provides advanced electronics that are optimized to produce clean and robust signals. Its data transmission rate is 128 samples per second, which provides the ability to perform an in-depth analysis on the brain activity. It has a minimum voltage resolution of 0.51 volts least significant bit (LSB) with 5 EEG electrodes at AF3,AF4,T7,T8,Pz locations and 2 reference electrodes. The headset is shown in Figure 3 with the five electrodes highlighted for reference. The device uses 14 bits for quantization, where 1 LSB = 0.51 μV. A 16-bit analog to digital conversion (ADC) is used, where 2 bits of instrumental noise floor are discarded. The reference electrodes CMS/DRL were located on left mastoid bone. The participants were asked to close their eyes for a duration of three minutes and were instructed to keep their head still to reduce movement artifacts. This also helped in minimizing the muscular motion and reduce these artifacts, since we recorded data at the frontal electrodes. A closed-eye condition was used, since correlates of long-term stress have been found in this condition in previous studies [10,33]. Another advantage of using the closed eye condition is the minimization of eye blink artifact. EEG signal acquisition was performed using the EMOTIV Xavier TestBench v.3.1.21. EEG signals were recorded from the scalp of participants while they were seated in a comfortable chair. Our experiments were specifically carried out in the afternoon (between 3–5 pm) to comply with similar studies where the circadian rhythm was assumed to be similar at this time period for the participants. 

### 3.2. Pre-Processing

The EEG signals recorded from the scalp contained noise due to external interference. Before feature extraction, noise was removed from the signals for better classification results. The data recorded using the EEG electrodes provided by Emotiv have a DC offset in their value that should be removed before doing analysis based on fast Fourier transform. The average value of data from each channel was subtracted from the sample values to remove the DC offset. For reducing muscular artifacts, participants were instructed to minimize their head movements during the EEG acquisition. In a closed eye condition, blink artifacts were also found to be minimal. EMOTIV Insight has a frequency response of 1–43 Hz, which makes the signal noise free from AC line interference at 50 Hz.

### 3.3. Feature Extraction and Selection

Neural oscillatory features are widely used in literature for EEG-based classification systems. EEG signals are decomposed into different frequency bands. The Welch method was used to extract power spectral densities with a window length of 128 samples with 50 percent overlap. For feature extraction, power spectral densities of different neural oscillations namely, delta (1–3 Hz), theta (4–7 Hz), alpha (8–12 Hz), beta (13–30 Hz), gamma (25–43 Hz), slow (4–13 Hz), and low beta (13–17 Hz) were computed from each channel. Relative gamma waves were computed by taking the ratio of slow and gamma waves. Eight features from each of the five channels adds up to forty features. Moreover, five alpha and beta asymmetries were calculated, giving a total of forty-five neural oscillatory features. The alpha asymmetries were calculated using the following equations,

(1) $$\alpha_{f}=\frac{\alpha_{AF4}-\alpha_{AF3}}{\alpha_{AF3}+\alpha_{AF4}},$$

(2) $$\alpha_{t}=\frac{\alpha_{T8}-\alpha_{T7}}{\alpha_{T8}+\alpha_{T7}},$$

(3) $$\alpha_{a}=\alpha_{f}+\alpha_{t},$$

where $\alpha_{f},\alpha_{t}$ and $\alpha_{a}$ represents the frontal alpha, temporal alpha, and alpha asymmetry respectively and $\alpha_{channel}$ represents the alpha power spectral density of the frontal and temporal EEG channels. Similarly, the frontal and temporal beta asymmetries were calculated using,

(4) $$\beta_{f}=\frac{\beta_{AF4}-\beta_{AF3}}{\beta_{AF3}+\beta_{AF4}},$$

(5) $$\beta_{t}=\frac{\beta_{T8}-\beta_{T7}}{\beta_{T8}+\beta_{T7}},$$

where $\beta_{f}$, and $\beta_{t}$ represents the frontal and temporal beta asymmetries and $\beta_{channel}$ represents the beta power spectral densities for the frontal and temporal EEG channels. Features were selected using a *t*-test for determining the statistical significance of features in stress and control group. A lower *p*-value returned by the *t*-test shows that the feature was significantly discriminating in stress and control group.

### 3.4. Subject Labeling

The proposed method uses two types of labeling for supervised classification. PSS-10 was used for the questionnaire-based labeling method to subjectively evaluate the stress of participants. This questionnaire consists of ten questions. Each question asks the subject about the frequency of stressful events that have occurred during a period covering the last thirty days. The response for each question is on a scale of 0 to 4, where 0 represents that the event never occurred and 4 represents a frequent occurrence. The total PSS-10 score for each participant has a range between 0 and 40. The participants are divided in two groups i.e., the control and stress group, using the PSS score. A threshold was selected for this purpose, which was given by the following equation,

(6) $$T_{p}=\mu \pm \frac{\sigma }{2},$$

where $T_{p}$ is threshold of PSS score, μ is the mean, and σ is standard deviation of the PSS scores.

The psychologist assigned labels for the stress and control groups after an expert evaluation based on the interview and PSS scores. During the interview, the expert investigated the physical, emotional, behavioral, and cognitive symptoms of stress. Physical symptoms included aches or pain, diarrhea or constipation, nausea, dizziness, chest pain, and rapid heart rate. Emotional symptoms of stress included depression, anxiety, moodiness, irritability, overwhelming feelings, and loneliness. Behavioral and cognitive symptoms included memory problems, inability to concentrate, poor judgment, negativity, racing thoughts, and constant worrying. The interviews were conducted by the psychologist who was affiliated with a public sector hospital. The labels (control/subject) were assigned to participants by the expert based on the responses and the PSS score for each participant. The eighteen symptoms evaluated by the expert are presented in Table 1. The assigned labels were used as ground truth for training the system using the corresponding EEG recordings for each subject.

### 3.5. Stress Classification

In this study, five different types of classifiers were used for classification, which are described in the following subsections very briefly to make the manuscript self contained.

#### 3.5.1. Support Vector Machine

A support vector machine uses the statistical learning theory based on the principle of structural risk minimization. An SVM selects a hyper-plane, which separates the feature space in to control and stress group according to the labels provided. The SVM is a highly efficient classifier and is used widely for stress classification in EEG based studies [19,25]. The use of SVM reduces the risk of data over-fitting and provides good generalization performance.

#### 3.5.2. The Naive Bayes

Naive Bayes is a probabilistic classifier based on Bayes theorem. It uses the maximum posterior hypothesis of statistics and works well for high dimensional input data. It is a nonlinear classifier and gives good results in real world problems. In addition, the Naive Bayes classifier requires a small amount of training data to approximate the statistical parameters [36].

#### 3.5.3. K-Nearest Neighbors

KNN is an instance-based learning classifier, where training instances are stored in their original form. A distance function is used to determine the member of the training set, which is nearest to a test example and used to predict the class. The distance function is easily determined if the attributes are numeric. Most instance-based classifiers use Euclidean distance for distance calculation. The distance between an instance with attribute values $a_{1},a_{2},\ldots ,a_{n}$ (where n is the number of attributes) and $b_{1},b_{2},\ldots ,b_{n}$ is defined as,

(7) $$D_{g}=\sqrt{{\left(a_{k}-b_{k}\right)}^{2}}.$$

#### 3.5.4. Logistic Regression

The logistic regression algorithm guards against over-fitting by penalizing large coefficients. The output is set to one for training instances belonging to the class and zero otherwise. Logistic regression builds a linear model based on a transformed target variable, where a transformation function converts a nonlinear function to a linear function.

#### 3.5.5. Multi-Layer Perceptron

In a multi-layer perceptron structure, transfer functions are used for mapping inputs to the output. These functions include sigmoid function, rectified linear unit, and hyperbolic tangent. The classifier uses back-propagation to classify instances. Multi-layer perceptrons are trained by minimizing the squared error of the network output, essentially treating it as an estimate of the class probability, which is given by the following equation,

(8) $$E=\frac{1}{2}\left(\left(y-f{\left(x\right)}^{2}\right)\right),$$

where f(x) is the network prediction obtained from the output unit and *y* is the instance class label.

## 4. Results and Discussion

### 4.1. Dataset

A total of 33 participants related to the education field volunteered for this study. The participants reported no history of brain injury and they were not using any medications that could have affected their brain activity at the time of experiment. Among these 33 healthy participants, 20 were male and 13 were females (60.6% male and 39.4% female). The participant’s ages ranged from 18 to 40 years (μ=23.85, SD=5.48). In line with the Helsinki Declaration [37] and the departmental ethics guidelines, all participants of the study were briefed about the research goals. In addition, a signed informed consent was obtained from each participant. This study was approved by the Directorate of Advanced Studies and Research at the University of Engineering and Technology, Taxila.

### 4.2. Performance Parameters

The parameters used in this study include average accuracy rate, Kappa statistic, F-measure, mean absolute error (MAE), and root mean absolute error (RMAE). Accuracy is the ratio of truly classified instances over total number of instances in the recorded data. F-measure is calculated by considering the precision and recall values. The Kappa statistic values ranges between 0 and 1, where 0 represents chance level classification and 1 means perfect classification. A value less than zero shows that the classification is worse than chance level. For stress classification, the generalization performance of the proposed system was tested using cross validation to avoid over- and under-fitting as well as to make sure the the proposed system adopts well to unseen data. A 10-fold cross validation technique was used in this study, where the training data was randomly divided into ten equal parts (nine parts for train and one part for test) and the process was repeated 10 times. During the process, every instance was used for testing at a time and the remaining instances were used for training of the classifier.

### 4.3. Stress and Control Group

**Figure 4:**
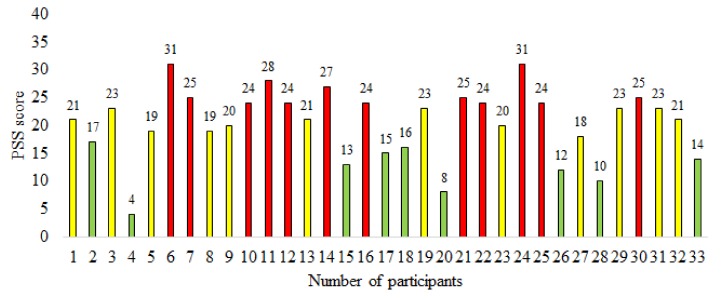
A graphical representation of Perceived Stress Scale (PSS) scores for participants showing labels assigned using the PSS based labeling method (green: control group, red: stress group, yellow: neutral).

The scores acquired from participants using the PSS questionnaire are shown in Figure 4. The green and red bars represent the PSS scores of participants belonging to the control and stress groups respectively. The yellow bars indicate the PSS scores of participants not considered in either the stress or the control group. Overall, for the PSS scores we have (μ,σ)=(20.4±6.14). A participant with a PSS score below 17.33 was considered to be in control group, whereas a participant with a PSS score higher than 23.47 was categorized in the stress group. These values were calculated using the threshold criteria defined in Equation (6). Hence 12 participants were put into the stress group (red bars) and 9 into the control group (green bars).

In expert (hybrid) evaluation, the psychology expert considered both PSS scores and the symptoms obtained from the interview method. The expert interviewed each participant for an average duration of 25 min. Out of the 33 participants, 10 were assigned to the stress group and 10 were assigned to the control group. The details about each participant regarding gender, age, PSS score, the label assigned by using PSS score, and the label assigned by expert is given in Table 2. There were fifteen differences in the assigned labels between those assigned using PSS scores and the expert (hybrid) evaluation. The experimental results show that expert (hybrid) labeling helps in improving the classification of long-term stress. It is important to note here that in a majority of the cases regarding label mismatch (13 out of 15), the PSS score ranges between 17 and 25, which covers the neutral range. Since we hypothesize that the expert (hybrid) labeling is better suited for the classification task, we have used these labels as ground truth.

### 4.4. Feature Selection Using t-Test

We used a two-sided Student’s *t*-test with a significance level of 0.05 and results using the *p*-values are shown in Table 3 for different EEG oscillations. For the *t*-test, the degree of freedom was 9 and the null hypothesis was tested for various features for stress and control groups. It is evident that at a confidence level of 0.05, none of the extracted feature were found statistically significant in the stress and control condition when PSS-based labeling was used for the reference standard. It is also revealed that beta and gamma waves from AF3 are statistically significant features in the stress and control group, when labels assigned by expert evaluation were used as a reference standard. Five additional features, namely frontal ($\alpha_{f}$) and temporal ($\alpha_{t}$) alpha asymmetries, frontal ($\beta_{f}$) and temporal ($\beta_{t}$) beta asymmetries, and alpha asymmetry ($\alpha_{a}$) were also used (see Equations (1)–(5)). Results of the *t*-test applied over these features in stress and control groups are presented in Table 4. It can be seen that alpha asymmetry is statistically different between the stress group and the control group using expert-based labeling. Three significant features, namely beta (AF3), gamma (AF3), and alpha asymmetry were selected for long-term stress classification based on the results of *t*-test. A *p*-value of 0.04 and 0.03 for beta and gamma oscillations indicated their statistical significance. The *p*-value of alpha asymmetry from frontal and temporal channels was 0.0005, indicating the statistical significance of alpha asymmetry from both temporal and frontal regions.

**Figure 5:**
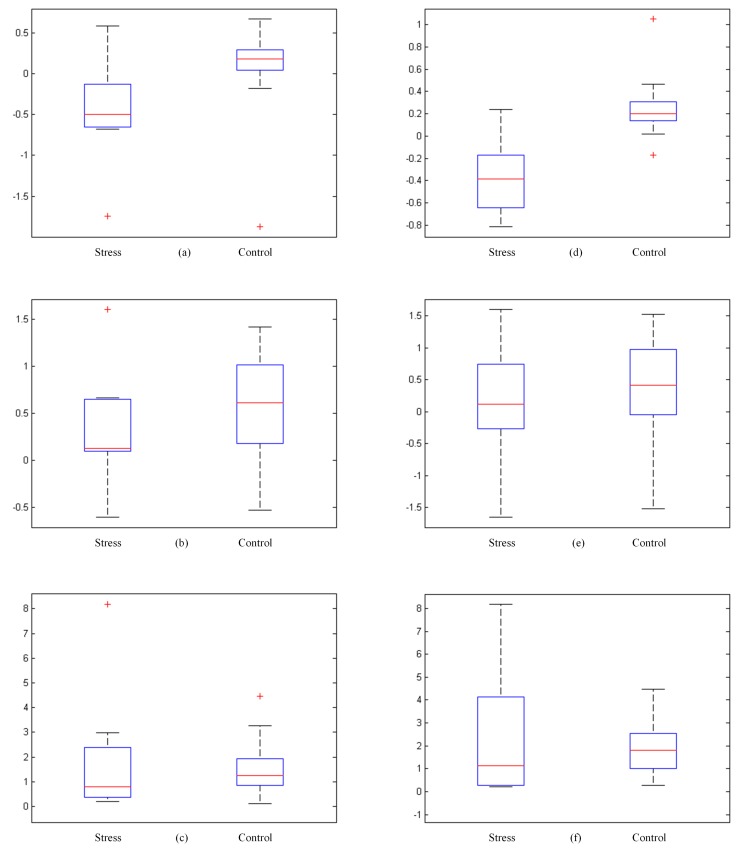
Box plots of features. (**a**) Alpha asymmetry; (**b**) beta; (**c**) gamma; (**d**) alpha asymmetry (EE); (**e**) beta (EE); (**f**) gamma (EE); EE represents the labeling method of expert evaluation.

The box plots are presented in Figure 5, where the first row represents features acquired through PSS labeling including alpha asymmetry, beta, and gamma respectively. The second row shows the same features acquired through expert evaluation. The + indicates an outlier, and the red line within the box represents the median value. A comparatively short box plot suggests that the features are in agreement with each other. A taller box plot suggests features show different distribution within themselves. From box plots (Figure 5b,c,e,f) it is observed that there is not much difference in the beta and relative gamma features to differentiate stress and control groups for both expert- and PSS-based labels. However, Figure 5a,d are candidates for good features as they appear to differentiate the stress and control group. In Figure 5a, the alpha asymmetry for the stressed group does not have a long lower whisker, which shows alpha asymmetry is not varied along the negative quartile, while in Figure 5d, the stressed group has varied alpha asymmetry as shown by the lower and upper whiskers. Also, in Figure 5d the median is comparatively at the center of the distribution. This suggests that alpha asymmetry is a good candidate to be used in the stress classification task.

### 4.5. Classification

We performed a comprehensive set of experiments to test and validate our proposed model using five classifiers, namely, KNN, NB, SVM, LR, and MLP. These classifiers were used with alpha asymmetry, beta, and gamma waves from channel AF3 as features to classify long-term stress. Each combination of the selected features was analyzed with each of the classifiers. The results of these classifiers in terms of average accuracy are shown in Table 5. We used 10-fold cross validation in these experiments since our dataset was limited. We used 10 folds, where in each fold 90% of the data were used for training and 10% for testing and reporting the average values of parameters across all 10 folds. The hyper parameters for classifiers used in our experiment were chosen using a grid search.

We observed that the classifier accuracy was high whenever alpha asymmetry was either used as a single feature or in combination with other features. The SVM- and LR-based classifiers give the highest accuracy when alpha asymmetry was used as a feature. The performance evaluation parameters for these classifiers are given in Table 6. We also observed that both SVM and LR show very similar values for kappa statistic and F-measure. SVM may have a slightly lesser mean absolute error of 0.15 than that of logistic regression with a value of 0.22, whereas LR has a lesser RMAE of 0.36 than that of SVM i.e., 0.38. The overall classification accuracy of both these classifier is similar. Overall, we concluded that SVM may be a better choice for an assisting system for stress recognition.

### 4.6. Discussion

Numerous studies have analyzed brain activities under stressful conditions, which are induced by a task such as impromptu speech, examination, mental task, public speaking, and the cold pressor test [38,39,40,41,42,43]. These studies evaluate short-term induced stress, whereas the classification of long-term stress using EEG has not been widely investigated. In Table 7, studies involving EEG to classify human stress are presented for comparison. It is observed that different stress-inducing tasks were used such as driving simulation, examination, and mental arithmetic tasks. Specialized instruments like MIST and Stroop tests were also used to induce stress. For chronic stress there could be several stressors that affect the physical, emotional, cognitive, or behavioral well being of a human being. Therefore, it is proposed that recording resting state EEG for stress classification is a better choice without involving stress induction. The number of participants involved in such studies vary from 5 to 42. The SVM and NB were used as classifiers in most of the studies. SVM was found to be the most efficient classifier, giving a maximum accuracy of 96%, when stress was induced by mental arithmetic test. In [10], the resting state EEG was recorded for two minutes and a nonlinear analysis was performed but no classification algorithm was used. In [44], chronic stress has been classified with an accuracy of 90%, using EEG recordings from eight electrodes and a stress-inducing condition.

Despite the difficulties of EEG in stress studies, there are cases where the use of EEG is vital and it has a clinical meaning in various conditions. For instance, ECG is not a direct stress measurement system, especially when mental stress originates in the brain. Furthermore, we studied long-term stress, and we did not have any stress inducer in our study (unlike other ECG- and HRV-based studies); hence, EEG can be a modality of choice for our experiments and we show its effectiveness with our experimental results. Although EEG has not been widely used for long term stress classification in clinical practice, our proposed method attempts to establish this approach. It has been shown that conditions such as anxiety, tension, and depression decrease as the frontal asymmetry shifts to the right hemisphere of the brain giving significance to EEG laterality [22]. It was demonstrated that variations in the beta activity [31] and pre-frontal gamma [34] contribute towards stress assessment. Hence there is evidence suggesting that these oscillations in the pre-frontal brain region can be used for assessment of stress using EEG recordings.

It is shown in this study that the alpha asymmetry of the brain can be considered as a potential marker for the recognition of chronic stress in humans. We observed (Table 5) that the classification accuracy using beta and gamma oscillations was lower when compared to alpha asymmetry. Whenever a combination of alpha asymmetry from beta and gamma oscillations was used, the decision boundaries were changed. Due to this, the classification accuracy was lower when compared to the case when alpha asymmetry was individually used as a feature. The labeling should be performed by using a hybrid method (psychology expert and PSS scores) for training the system in a supervised manner. Due to the limited size of the data, we have shown that MLP is the only class of neural network based classifier that can fit to the task of stress classification. For more deeper networks, we would need more instances of EEG recordings.

## 5. Conclusions

In this paper, two different labeling methods were used for the classification of long-term stress in humans using EEG signals. Forty-five signal features were analyzed for the classification of chronic stress, and alpha asymmetry was found to be a discriminating feature when using expert’s evaluation as ground truth. The PSS scores, when used solely for labeling, returned no significant features. Furthermore, it is evident from our experimental results that SVM and LR give the highest accuracy (85.20%) for classification. We also observed that the stress group was better classified when compared to the control group irrespective of the classifiers used. Finally, we established that alpha asymmetry can be used a potential bio-marker for the classification of long-term stress with SVM. To the best of our knowledge, no previous EEG-based studies have involved a psychology expert for labeling of groups for long-term stress assessment. In the future, more features and participants will be considered for the analysis. With the availability of more data, deep learning based strategies can be applied for potentially improved methods. 

## Figures and Tables

**Table 1:** The symptoms evaluated by expert psychologist during the interview process.

Sr. No.	Symptom	Type of Symptom
1	Aches and pains	Physical
2	Diarrhea or constipation	Physical
3	Nausea & Physical pain	Physical
4	Dizziness	Physical
5	Chest pain	Physical
6	Rapid heart beat	Physical
7	Depression or general happiness	Emotional
8	Anxiety or Agitation	Emotional
9	Moodiness	Emotional
10	Irritability	Emotional
11	Feeling overwhelmed	Emotional
12	Loneliness and isolation	Emotional
13	Memory problems	Behavioral and Cognitive
14	Inability to concentrate	Behavioral and Cognitive
15	Poor judgment	Behavioral and Cognitive
16	Seeing only the negative	Behavioral and Cognitive
17	Anxious or racing thoughts	Behavioral and Cognitive
18	Constant worrying	Behavioral and Cognitive

**Table 2:** Gender, age, PSS score, and labels for the participants according to PSS and expert-based (hybrid) labeling. (A-control group, B-stress group, X-neutral).

Participant No.	Gender	Age	PSS Score	PSS Label	Expert Label
1	M	28	21	X	X
2	M	29	17	A	X
3	M	23	23	X	X
4	M	32	4	A	A
5	F	19	19	X	A
6	F	18	31	B	B
7	M	24	25	B	X
8	M	33	19	X	A
9	M	21	20	X	B
10	M	22	24	B	X
11	F	20	28	B	B
12	M	19	24	B	B
13	M	24	21	X	A
14	F	20	27	B	B
15	M	23	13	A	X
16	M	21	24	B	X
17	F	19	15	A	A
18	M	25	16	A	A
19	F	21	23	X	B
20	M	34	8	A	A
21	M	33	25	B	X
22	F	21	24	B	B
23	M	31	20	X	B
24	F	24	31	B	B
25	F	20	24	B	B
26	M	19	12	A	A
27	M	21	18	X	A
28	M	21	10	A	X
29	F	21	23	X	X
30	F	23	25	B	X
31	M	20	23	X	X
32	M	40	21	X	X
33	F	20	14	A	A

**Table 3:** Results for the *t*-test on various neural oscillations including PSS and expert-based labeling methods.

Labeling Method	Neural Oscillations								
	Channel	delta (δ)	theta (θ)	slow	alpha (α)	$\beta_{l}$	beta (β)	gamma (γ)	RG
PSS	AF3	0.12	0.09	0.13	0.28	0.30	0.21	0.32	0.53
	T7	0.89	0.81	0.61	0.21	0.58	0.85	0.52	0.36
	Pz	0.15	0.16	0.16	0.19	0.29	0.46	0.64	0.30
	T8	0.89	0.97	0.95	0.87	0.49	0.97	0.90	0.26
	AF4	0.14	0.12	0.13	0.22	0.20	0.15	0.23	0.79
Expert	AF3	0.65	0.50	0.51	0.08	0.95	**0.04**	**0.03**	0.23
	T7	0.92	0.60	0.51	0.15	0.99	0.42	0.54	0.99
	Pz	0.91	0.89	0.90	0.90	0.93	0.69	0.34	0.40
	T8	0.54	0.51	0.55	0.48	0.85	0.96	0.85	0.56
	AF4	0.11	0.12	0.12	0.35	0.25	0.21	0.28	0.61

**Table 4:** Asymmetries in PSS and expert evaluation.

Features	$\alpha_{t}$	$\alpha_{f}$	$\beta_{t}$	$\beta_{f}$	$\alpha_{a}$
PSS	0.23	0.39	0.91	0.45	0.11
Expert	0.21	0.07	0.49	0.73	0.0005

**Table 5:** Accuracy of classifiers for various combinations of statistically significant features.

Features	SVM	NB	KNN	LR	MLP
$\alpha_{a}$	85.20	80.11	65.32	85.15	80.12
γ	70.32	50.21	50.43	50.33	50.17
β	55.07	50.01	50.51	50.48	50.70
β, γ	70.45	50.65	50.09	50.65	50.02
$\alpha_{a}$,β	85.15	80.02	65.38	85.04	85.01
$\alpha_{a}$, γ	80.91	80.79	65.55	85.08	85.05
$\alpha_{a}$, β, γ	80.83	80.77	65.96	85.09	85.13

**Table 6:** Evaluation parameters for the best performing classifiers with $\alpha_{a}$ as a feature.

Classifier	Average Accuracy	Kappa	F-Measure	MAE	RMAE
LR	85.15	0.70	0.85	0.22	0.36
SVM	85.20	0.71	0.87	0.15	0.39

**Table 7:** Comparison of results with previously related EEG-based studies.

Related Work	Stress Inducer	Participants	Classifier	Accuracy
Lin et. al. [45]	Driving simulator	6	KNN and **NBC**	71.77
Vijean et. al. [46]	Mental arithmetic task	5	NN	91.17
Khosrowabadi et. al. [44]	Examination	26	KNN and SVM	90.00
Jun et. al. [47]	Arithmetic task and stroop test	10	SVM	96.00
Al-Shargie et. al. [19]	Mental arithmetic task	18	SVM and **ECoC**	95.37
Subhani et. al. [25]	MIST	42	LR, SVM and NB	94.60
Saeed et. al. [33]	None	28	NB	71.43
**Proposed**	**None**	**33**	**SVM**	**85.20**

## References

[1] Selye H. (1965). The stress syndrome. Am. J. Nurs..

[2] Heim C., Nemeroff C.B. (2002). Neurobiology of early life stress: Clinical studies. Semin. Clin. Neuropsychiatry.

[3] McGonagle K.A., Kessler R.C. (1990). Chronic stress, acute stress, and depressive symptoms. Am. J. Commun. Psychol..

[4] Cohen S., Janicki-Deverts D., Miller G.E. (2007). Psychological stress and disease. JAMA.

[5] Steptoe A., Kivimäki M. (2012). Stress and cardiovascular disease. Nat. Rev. Cardiol..

[6] Van Praag H. (2004). Can stress cause depression?. Prog. Neuro-Psychopharmacol. Biol. Psychiatry.

[7] Hammen C., Dalton E.D., Thompson S.M. (2014). Measurement of chronic stress. Encycl. Clin. Psychol..

[8] Sobell L.C., Toneatto T., Sobell M.B., Schuller R., Maxwell M. (1990). A procedure for reducing errors in reports of life events. J. Psychosom. Res..

[9] McQuaid J.R., Monroe S.M., Roberts J.R., Johnson S.L., Garamoni G.L., Kupfer D.J., Frank E. (1992). Toward the standardization of life stress assessment: Definitional discrepancies and inconsistencies in methods. Stress Med..

[10] Peng H., Hu B., Zheng F., Fan D., Zhao W., Chen X., Yang Y., Cai Q. (2013). A method of identifying chronic stress by EEG. Pers. Ubiquitous Comput..

[11] Zheng R., Yamabe S., Nakano K., Suda Y. (2015). Biosignal analysis to assess mental stress in automatic driving of trucks: Palmar perspiration and masseter electromyography. Sensors.

[12] Ahn J.W., Ku Y., Kim H.C. (2019). A Novel Wearable EEG and ECG Recording System for Stress Assessment. Sensors.

[13] Mehreen A., Anwar S.M., Haseeb M., Majid M., Ullah M.O. (2019). A Hybrid Scheme for Drowsiness Detection using Wearable Sensors. IEEE Sens. J..

[14] Asif A., Majid M., Anwar S.M. (2019). Human stress classification using EEG signals in response to music tracks. Comput. Biol. Med..

[15] Saeed U., Muhammad S., Anwar S.M., Majid M., Awais M., Alnowami M. (2018). Selection of Neural Oscillatory Features for Human Stress Classification with Single Channel EEG Headset. BioMed Res. Int..

[16] Raheel A., Anwar S.M., Majid M. (2018). Emotion recognition in response to traditional and tactile enhanced multimedia using electroencephalography. Mult. Tools Appl..

[17] Anwar S., Saeed S., Majid M., Usman S., Mehmood C., Liu W. (2018). A Game Player Expertise Level Classification System Using Electroencephalography (EEG). Appl. Sci..

[18] Sanei S., Chambers J.A. (2007). EEG Signal Processing.

[19] Al-shargie F., Tang T.B., Badruddin N., Kiguchi M. (2018). Towards multilevel mental stress assessment using SVM with ECOC: An EEG approach. Med. Biol. Eng. Comput..

[20] Fisch B. (1999). Fisch and Spehlmann’s EEG Primer: Basic Principles of Digital and Analog EEG.

[21] Davidson R.J. (2004). What does the prefrontal cortex “do” in affect: Perspectives on frontal EEG asymmetry research. Biol. Psychiatry.

[22] Papousek I., Schulter G. (2002). Covariations of EEG asymmetries and emotional states indicate that activity at frontopolar locations is particularly affected by state factors. Psychophysiology.

[23] Lobo I., Portugal L.C., Figueira I., Volchan E., David I., Pereira M.G., de Oliveira L. (2015). EEG correlates of the severity of posttraumatic stress symptoms: A systematic review of the dimensional PTSD literature. J. Affect. Disord..

[24] Goncharova I.I., Barlow J.S. (1990). Changes in EEG mean frequency and spectral purity during spontaneous alpha blocking. Electroencephalogr. Clin. Neurophysiol..

[25] Subhani A.R., Mumtaz W., Saad M.N.B.M., Kamel N., Malik A.S. (2017). Machine learning framework for the detection of mental stress at multiple levels. IEEE Access.

[26] Cai H., Han J., Chen Y., Sha X., Wang Z., Hu B., Yang J., Feng L., Ding Z., Chen Y. (2018). A Pervasive Approach to EEG-Based Depression Detection. Complexity.

[27] Baghdadi A., Aribi Y., Alimi A.M. Efficient Human Stress Detection System Based on Frontal Alpha Asymmetry. Proceedings of the 24th International Conference, ICONIP 2017.

[28] Aspinall P., Mavros P., Coyne R., Roe J. (2015). The urban brain: analysing outdoor physical activity with mobile EEG. Br. J. Sports Med..

[29] Düsing R., Tops M., Radtke E.L., Kuhl J., Quirin M. (2016). Relative frontal brain asymmetry and cortisol release after social stress: The role of action orientation. Biol. Psychiatry.

[30] Kaiser A.K., Doppelmayr M., Iglseder B. (2018). Electroencephalogram alpha asymmetry in geriatric depression. Zeit. Für Geront. Und Ger..

[31] Seo S.H., Lee J.T. (2010). Stress and EEG. Convergence and Hybrid Information Technologies.

[32] Gärtner M., Grimm S., Bajbouj M. (2015). Frontal midline theta oscillations during mental arithmetic: Effects of stress. Front. Behav. Neurosci..

[33] Saeed S.M.U., Anwar S.M., Majid M. (2017). Quantification of human stress using commercially available single channel EEG Headset. IEICE Trans. Inf. Syst..

[34] Minguillon J., Lopez-Gordo M.A., Pelayo F. (2016). Stress assessment by prefrontal relative gamma. Front. Comput. Neurosci..

[35] Arsalan A., Majid M., Butt A.R., Anwar S.M. (2019). Classification of Perceived Mental Stress Using a Commercially Available EEG Headband. IEEE J. Biomed. Health Inform..

[36] Kotsiantis S.B., Zaharakis I., Pintelas P. (2007). Supervised machine learning: A review of classification techniques. Emerg. Artif. Intell. Appl. Comput. Eng..

[37] Association W.M. (2014). World Medical Association Declaration of Helsinki: Ethical principles for medical research involving human subjects. J. Am. Coll. Dent..

[38] Knaus J., Wiese R., Janßen U. The processing of word stress: EEG studies on task-related components. Proceedings of the 16th International Congress of Phonetic Sciences.

[39] Matsunami K., Homma S., Han X.Y., Jiang Y.F. (2001). Generator sources of EEG large waves elicited by mental stress of memory recall or mental calculation. Jpn. J. Phys..

[40] Lewis R.S., Weekes N.Y., Wang T.H. (2007). The effect of a naturalistic stressor on frontal EEG asymmetry, stress, and health. Biol. Psychiatry.

[41] Seo S., Gil Y., Lee J. The relation between affective style of stressor on EEG asymmetry and stress scale during multimodal task. Proceedings of the Third International Conference on Convergence and Hybrid Information Technology, CCIT’08.

[42] Miller P.F., Light K.C., Bragdon E.E., Ballenger M.N., Herbst M.C., Maixner W., Hinderliter A.L., Atkinson S.S., Koch G.G., Sheps D.S. (1993). Beta-endorphin response to exercise and mental stress in patients with ischemic heart disease. J. Psychiatr. Res..

[43] Hassellund S.S., Flaa A., Sandvik L., Kjeldsen S.E., Rostrup M. (2010). Long-term stability of cardiovascular and catecholamine responses to stress tests: An 18-year follow-up study. Hypertension.

[44] Khosrowabadi R., Quek C., Ang K.K., Tung S.W., Heijnen M. A Brain-Computer Interface for classifying EEG correlates of chronic mental stress. Proceedings of the 2011 International Joint Conference on Neural Networks.

[45] Lin C.T., Ko L.W., Chiou J.C., Duann J.R., Huang R.S., Liang S.F., Chiu T.W., Jung T.P. (2008). Noninvasive neural prostheses using mobile and wireless EEG. IEEE.

[46] Vijean V., Hariharan M., Saidatul A., Yaacob S. Mental tasks classifications using S-transform for BCI applications. Proceedings of the 2011 IEEE Conference on Sustainable Utilization and Development in Engineering and Technology (STUDENT).

[47] Jun G., Smitha K. EEG based stress level identification. Proceedings of the 2016 IEEE International Conference on Systems, Man, and Cybernetics (SMC).

